# A high-fat and high-fructose diet lowers the cecal digesta’s weight and short-chain fatty acid level of a Sprague–Dawley rat model

**DOI:** 10.3906/sag-1911-14

**Published:** 2021-02-20

**Authors:** Etik SULISTYOWATI, Dian HANDAYANI, Setyawati SOEHARTO, Achmad RUDIJANTO

**Affiliations:** 1 Department of Nutrition, Malang State Health Polytechnic Ministry of Health, Malang Indonesia; 2 School of Nutrition, Faculty of Medicine, Brawijaya University, Malang, Indonesia; 3 Department of Pharmacology, Faculty of Medicine, Brawijaya University, Malang, Indonesia; 4 Department of Endocrinology, Faculty of Medicine, Brawijaya University, Malang, Indonesia

**Keywords:** Cecal digesta, diet, high-fat, high-fructose, short-chain fatty acid, SCFA

## Abstract

**Background/aim:**

This study aimed to analyze the effect of a high-fat and high-fructose diet (HFFD) on the digesta weight and short-chain fatty acid (SCFA) levels of cecal digesta in rats.

**Materials and methods:**

This study was an experimental study with a posttest-only control group design with male Sprague–Dawley strain rats as the samples. A total of 36 rats were divided into two groups with normal diet (N) and modified HFFD. The data of energy intake, nutrients and fiber, body weight, Lee index, abdominal circumference, digesta weight, and SCFA levels of cecal digesta were collected.

**Results:**

The results showed an 11.94% increase in body weights of rats with HFFD. The total energy intake of the HFFD group was significantly higher than that of N (p = 0.000). The fiber intake and cecal digesta weight in group N were higher than that in the HFFD group (p = 0.00 and p = 0.02, respectively). The concentrations of acetate, butyrate, propionate, and total SCFA in the N group were significantly higher than in the HFFD (p = 0.041,,p = 0.004, p = 0.040, p = 0.013, respectively). A significant negative relationship was observed between the abdominal circumference and cecal digesta concentration (p = 0.029; r = −0.529) and between the Lee index and the SCFA concentration of cecal digesta (p = 0.036, r = −0.206).

**Conclusion:**

The research results showed that HFFD can reduce the weight and SCFA concentration of the cecal digesta. The negative relationship between abdominal circumference, the Lee index, and the SCFA concentration indicates the potential role in obesity incidence and metabolic diseases.

## 1. Introduction

Obesity results from the imbalance between the energy spent and energy intake, which influences various metabolic pathways related to metabolites and hormones [1]. The number of people with obesity has increased, and it is predicted to increase up to 57.8%, especially for the adult population in the world in 2030 [2,3]. Current studies suggest that obesity is often accompanied by diseases such as insulin resistance, dyslipidemia, and hypertension and increases the risk of type 2 diabetes and cardiovascular diseases [4–7]. Based on the data from a basic health research (Riskesdas) in 2018, the prevalence of obesity increased from 14.8% in 2013 to 21.8% in 2018 in Indonesia [8].

Several studies explained that increased consumption of foods and beverages with high-fructose corn syrup would induce an increase in the obesity prevalence. Fructose can facilitate the glucose absorption but it will lead to malabsorption when the fructose content is higher than glucose. Fructose has a higher potential than sucrose to generate hepatic uric acid and triglycerides, which causes fatty liver [9]. Mamikutty et al. reported that rat subjects had obesity, hypertension, hyperglycemia, and hypertriglyceridemia after drinking water with 20% fructose for 8 weeks [10]. Fructose causes addiction and leptin resistance in the brain while also decreasing cholecystokinin expression and the growth hormone expression in the ventromedial nucleus. Long-term fructose consumption increases calorie intake due to loss of satiety signals in the brain, eventually resulting in obesity [11–13].

A low-fiber diet causes low short-chain fatty acid (SCFA) production by the intestinal microflora. SCFAs, such as acetate, propionate, and butyrate, are produced through fermentation by intestinal microflora from dietary fibers [11]. One of the roles of the intestinal microflora is to convert the free fatty acids (FFAs) from dietary fats to other FFAs as their metabolism results, which are the key factor in energy metabolism [14,15]. Dietary fiber has a beneficial metabolic effect on body weight, as well as glucose homeostasis, food intake, and insulin sensitivity [14,15].

SCFA involvement in energy and lipid metabolism has gained attention as the potential in controlling metabolic syndrome. Several studies showed that decreased obesity and insulin resistance occurred in the experimental animals with a high-fat diet after butyrate-containing food supplementation [12]. The mechanism that explains the higher fecal SCFA production in the obese population remains a subject of debate. It could be due to the increased dietary substrate intake or the consequence of the increased metabolic activity of a certain group of bacteria; interestingly, some studies show inconsistent results. For example, Fernandes et al. [16] showed experimental results that are contrary to the results of research conducted by Lin et al. [17], Weitkunat et al. [18], and Miyamoto et al. [19]. There was an increase in the diet, but there was also a decrease in SCFA concentrations.

The controversy over these results prompted researchers to conduct studies on obese rats with a high-fat high-fructose diet (HFFD) and measure the weight and concentration of SCFA in the cecum rather than in the feces. Therefore, the present study aims to analyze the effect of HFFD administration on digesta weight and SCFA levels in rat cecum, as only 5%–10% of SCFA is excreted in the feces [20]. Using the obesity animal model, several factors related to food can be controlled, and the concentration of SCFA in the cecum can be analyzed. Thus, this study provides more objective results that could be used as a basis for creating an experimental animal model of obesity and nutrition interventions. This study has several differences with the previous ones: (1) We used Sprague–Dawley rats while some studies used Wistar rats; (2) We used a high-fat high-fructose feed while previous studies used high-fat or high-carbohydrate feed; and (3) We measured the SCFA in cecal digesta while others measured it in the feces.

## 2. Materials and methods

This research was conducted from October 2017 until February 2018. The ethical approval for this research was obtained from the Faculty of Medicine, Universitas Brawijaya, Malang, Indonesia, No. 368/EC/KEPK/10/2017.

### 2.1. Materials

The feed was composed of corn starch, dextrin corn starch, sucrose, soybean oil, casein, egg white, agar, white butter, beef kidney fat, minerals (AIN-93 M-MX-Mineral Mix), vitamins (AIN-93 -VX-Vitamin Mix), L-cystine, and choline bitartrate.

### 2.2. Normal and HFFD diet formulation and feed energy and content analysis

The manufacturing of modified feed and analysis of energy content and nutrient of the feed were according to the research by Sulistyowati et al. [21]. Feed ingredients consist of corn starch, dextrin corn starch, sucrose, soybean oil, casein, egg white, gelatin, white butter, cow kidney fat, minerals (AIN-93 M-MX-Mineral Mix), vitamins (AIN-93 -VX-Vitamin Mix), L-cystine, and choline bitartrate. All ingredients are mixed together and formed as rat pellets. The compositions of dietary diet modification for the intervention in making animal models of obesity in this study are listed in Table 1 [21].

### 2.3. Animal preparation and experimental

#### 2.3.1. Experimental design

This study used a posttest-only control group design. The number of replications in this study was determined by the Federer formula [22]:


(1) (t-1)(n-1)≥15

where *t* is the number of the intervention group and *n* is the number of repetitions or the number of samples per group. The number of interventions was 2 in this study; therefore, the number of replications was at least 16. Then, a number of the backup experimental unit was added to anticipate unwanted possibilities, such as death. The correction to the number of replications was based on the Higgins formula:


(2) 1/(1-f)

with an estimated experimental unit dropout (f) of 10%, the number of subjects needed in this study was 36.

The white rats ((*Rattus norvegicus*) Sprague–Dawley strain) were obtained from the Animal Laboratory of Institut Pertanian Bogor. The rats had 200–250 g body weight, were aged 70–90 days postnatal, male, and healthy. Rats with abnormal motor movements, those that did not want to eat and drink, and those that had >10% weight loss during the adaptation period were excluded from the study. The Sprague–Dawley was chosen as an obese animal model because it is quieter and easier to handle. Besides the fact that the rats can show changes in metabolic status according to the research objectives, these rats also have a high survival rate [16,23–27].

Rat cages were placed in an approximately 2-cm-thick husk mat that was replaced every 3 days. Room temperature was maintained at 25 °C in 12 h of dark and light cycles. Rats were acclimated for 7 days, fed with normal rat feed, and given distilled water for drinking ad libitum. On the 7th day, 18 rats in the intervention group were given HFFD and 30% fructose solution drink for 12 weeks [23,24]. The administration of a normal diet and HFFD was conducted for 12 weeks. Maintenance and euthanasia of rats were done following the fixed procedures at the Institute of Biosciences of Universitas Brawijaya Malang.

#### 2.3.2. Sample collection and preparation

Cecal digesta sample preparation for the SCFA content and digesta weight analysis was carried out by cecal dissections. The contents of the cecum (digesta) were scraped off using tweezers and then weighed using an ABK 220-4M digital scale-Japan. Next, each of the digesta was stored at −20 °C for the SCFA examination.

### 2.4. Determination of parameters

#### 2.4.1. Calorie intake

The feed intake was calculated by subtracting the amount of the given feed (g) and the leftover (g) after 24 h. The same was applied to the drinks in the HFFD group.


(3) Feed intake=the provide feed (grams)-the leftover feed (grams)

Calorie intake was calculated by the amount of feed intake (g) and 30% fructose solution (mL) multiplied by the energy value of feed N, HFFD, and the 30% fructose solution.


(4) Calorie intake=feed intake (grams)×the energy density of the feed

(5) Total calorie=the feed calorie+the drinks calorie

#### 2.4.2. Body weight, length, abdominal circumference, and Lee index

Body weight measurements were carried out by weighing rats with a KERN 440-33N analytical balance (Kern & Sohn, Germany) once a week. The result was the average of the two weighing results. Body length was measured after the rat was anesthetized with ketamine-xylazine of 75–100 mg/kg + 5–10 mg/kg. The body length was measured from the tip of the nose to the anus or base of the tail (naso-anal). Abdominal circumference was also measured after the anesthesia. Abdominal circumference was measured circularly in the largest part of the abdomen. Body length and abdominal girth were measured using OneMed 235 metleen (OneMed, Indonesia) with an accuracy of 0.01 cm. Lee index was used to determine the degree of obesity:


(6) $$\text{Lee index}=\text{Body weight }{\left(\text{g}\right)}^{0,33}/\text{Body length }(\text{mm})\times 1000$$

#### 2.4.3. The weight of the cecal digesta

The weight of the cecal digesta was measured by using KERN ABJ 220-4M (Kern & Sohn, Germany) digital scales with an accuracy of 0.000 mg.

#### 2.4.4. SCFA levels

The digest samples stored at −20 °C were thawed and 4 mg of the samples were centrifuged at 10,000 rpm for 15 min. As much as 2 mL of supernatant was added into a 5-mL plastic tube and 30 mg of 5-sulphosalicylic acid was added. The solution was shaken, centrifuged at 3000 rpm for 10 min at 4 °C, and then filtered through a Millipore filter until the clear liquid was obtained. As much as 1 μL of supernatant was injected into the gas-liquid chromatography device (Chrompack CP 9002 series 946253, Netherlands) using a microsyringe. After 9 min, the area of the specified compound was drawn in the recorder paper. Before the sample was injected, a standard mixture of acetate, propionate, and butyrate was injected first with a concentration of 0.025%, 0.05%, 0.3%, and 0.5%, respectively. Then the regression equation was calculated, which reflected the relationship between the area of acetic acid, propionic, and butyrate standard (Y) with the concentrations of acetic acid, propionic, and butyrate standard (X) [16,28–31].

### 2.5. Data analysis

All observations and measurements were tabulated and made in the form of mean ± standard deviation (SD). The differences in the experimental animal characteristics, the weight of the cecum contents, and the concentration of SCFA from both groups were analyzed by using an independent t-test at a 95% confidence level. The correlation between variables was tested by a bivariate test and the relationship was determined using the Pearson’s correlation test. Data processing and analysis were done in Microsoft Excel and SPSS version 21 (IBM) for Windows.

## 3. Results

The characteristics of the initial subjects and the results of the interventions in this study are presented in Table 1. The rats had similar body weights at the beginning of the study as the mean initial body weights in both groups were not different (p = 0.945). After the HFFD dietary intervention for 12 weeks, there was an increase of 12% in the average body weight, higher than the normal but not statistically significant (p = 0.140). However, there was a significantly higher abdominal circumference in the HFFD compared to the N group (p = 0.009).

The mean digesta weight of rats with N feed was significantly higher than that of rats with HFFD feed (p = 0.02) (Figure). Meanwhile, the concentration of SCFA in cecal digesta rat with N feed was significantly higher than in rats with HFFD (Table 2).

Pearson’s correlation test results show a significant negative relationship between the abdominal circumference and SCFA concentration (r = −0.529; p = 0.029) while the negative relationship also showed between the Lee Index and the SCFA concentration of the cecal digesta (r = −0.204; p = 0.433). The relationships between abdominal circumference, Lee index, and SCFA concentration are presented in Table 3.

## 4. Discussion

Several studies explained that an increase in the prevalence of central obesity occurs along with the increase in consumption of foods and drinks containing high-fructose corn syrup (HFCS). HFCS is commonly used in a wide variety of favored food and beverage products such as soft drinks, pastries, cookies, gums, jelly, and desserts. In the long run, the addiction effects and leptin resistance due to the fructose increase the calorie intake because of the loss of satiety signals in the brain, which in turn, causes overweightness and obesity [26].

The intervention of the HFFD feed was recommended in producing experimental animal models of obesity [32]. Previous studies presented that the composition of the HFFD feed is 25%–35% carbohydrate, 50%–70% fat, 15%–25% protein, and 17%–30% fructose solution [23–25,33]. In this study, the composition of the HFFD feed was 20.51% carbohydrate, 57.57% fat, and 21.90% protein, with a fiber content of 24.25 g per 100 g of feed, and 30% fructose in drinks.

The results indicated that the modified AIN 93 HFFD did not provide significant changes in body weight. However, there was an 11.94% increase in body weight in the HFFD group. This result is in line with the previous studies that showed that the rat had moderate obesity if its body weight change was 10%–25% higher than those in the N group [13,34]. Those factors did not show a significant increase in the body weight because the modified feed formula still had a higher energy density than the N diet, about 4.21 Cal/g. According to Miras et al. [35], the average energy density for N feed-in obese rats’ models was <3.5 Cal/g. In this study, the high energy density of N feed was due to the use of carbohydrate source material so it could be easily molded as a mouse pellet [35]. The calorie intake in this study was not different from the result of Marques et al. [24], which was around 66 calories for the HFFD group and 51 to 53 calories for the N group. The calorie intake from the feed in the HFFD group was lower than in the N group; however, the total calories of the HFFD group were significantly higher than those of the N group. The high-calorie intake in the HFFD group was due to the drink that contained 30% fructose. Besides, the HFFD feed had less than 60% fat, which was one of the contributing factors [24].

Fiber intake in the N group was significantly higher than in HFFD. This was due to the lower fiber content in the HFFD feed. The HFFD feed generally has low carbohydrate content from dietary fiber [14]. It affected the weight of the digesta in the rat cecum. The weight of rat cecal digestion with the N diet was significantly higher than HFFD (p = 0.02). The increasing volume of feces is the effect of the dietary fiber’s metabolism in the digestive tract, especially in the colon [36].

Most of the metabolic functions of dietary fiber are related to the colon as the fiber is relatively unchanged in the stomach and small intestine. Bacterial flora works actively in the colon. Besides being used to increase the content and weight/volume of feces, the metabolic products are used to produce volatile fatty acids (acetate, butyrate, and propionate), which are the main anions in feces [36,37]. The data obtained from individuals who experienced sudden death showed that the SCFA concentrations in the cecum were around 131 mmol/kg lumen contents, ten times higher than those in the ileum (13 mmol/kg) [38,39].

The results of this study showed that the rats with N feed had significantly higher SCFA concentrations in cecal digesta than the HFFD (p = 0.013). The results showed a high-fiber diet with low fat caused a higher amount of SCFA feces compared with the low-fiber diet [40]. The majority of acetate is produced by enteric bacteria as a result of carbohydrate fermentation. Besides, one-third of colonic acetate is synthesized from hydrogen and carbon dioxide or formic acid through the Wood–Ljungdahl pathway by acetogenic bacteria [41,42]. Three different pathways used by colon bacteria for propionate formation are the succinate pathway, the acrylic pathway, and the propanediol pathway [43]. Fermented food fiber with cellulose substrate gives a proportion of 81% of acetate, 13% of propionates and 6% of butyrate. In the fermentation process in the large intestine, cellulose and pectate will produce the largest proportion of acetic acid, while hemicellulose potentially produces propionate acid [44]. This study showed that the amount of produced acetate was higher than propionate and butyrate was the least in quantity.

The results also showed the negative relationship between the increased abdominal circumference, which showed the degree of visceral obesity, with the decreased concentrations of cecal digesta (r = −0.529; p = 0.029); which was also similar to the Lee index with the decrease in the SCFA concentration in the cecal digesta. (r = −0.204; p = 0.433). These were consistent with Heinritz et al.’s study [31], which explained that SCFA concentrations were significantly higher in the administration of low-fat diets than high-fat; the significance values for acetate, butyrate, and propionate were p = 0.023, p = 0.013, and p = 0.003, respectively.

Similar results were also found by Barczynska et al. [45], who observed the SCFAs concentration in the feces of obese children was lower than normal-weight children (p = 0.04). It can be explained that the SCFA protective effect on the metabolic changes induced by a high-fat diet depends on the regulation of proliferator-active peroxisomes (PPARγ); it triggers the changes of lipid synthesis to lipid oxidation [46]. Interestingly, although the three intestinal SCFAs have a protective effect on obesity, butyrate and propionate are likely to have a more significant effect than acetate [47]. Different mechanisms have been proposed to explain this effect; one of them is the activation of signaling pathways mediated by protein kinases, such as adenosine monophosphate-activated protein kinases [12,46] or mitogen-activated protein kinases [48]. It has been reported that butyrate and propionate induce the intestinal hormone production, which can reduce food intake [47]. Acetate also has been proven to reduce appetite through interactions with the central nervous system [49]. One mechanism underlying the effect of SCFA on food intake and satiety is related to the release of intestinal hormone related to satisfaction, especially GLP-1 and the peptide YY (PYY). These proteins are secreted by intestinal L cell enteroendocrine, which is found in the distal ileum and colonic epithelium [50,51]. PYY affects appetite and satiety by suppressing neuropeptide Y (NPY) and activating the proopiomelanocortin (POMC) neurons in the hypothalamus or by delaying the gastric emptying process [52]. In addition to its role as incretin, GLP-1 also regulates the appetite by affecting POMC and NPY neurons in the hypothalamus which also inhibits gastric emptying and gastric acid secretion [53–56]. Unfortunately, the secretion from intestinal hormones (PYY and GLP-1) was not assessed in this study, which became a limitation in this study.

In conclusion, the administration of HFFD showed a weight and SCFA concentration reduction in the cecum contents, which is harmful to health. However, further experimental studies are still needed to get more deep information before it can be applied to the human diet.

## Figures and Tables

**Figure:**
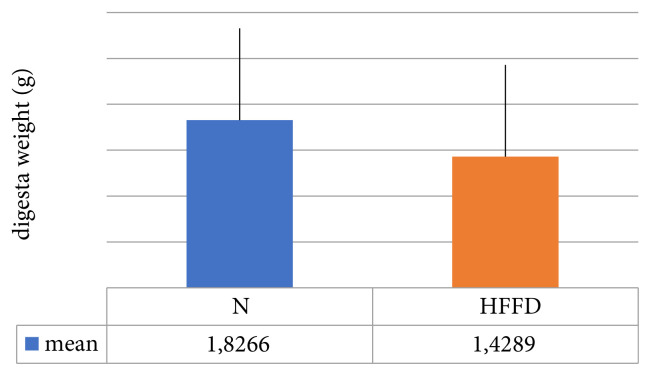
The average weight of cecal digesta by group.

**Table 1:** Characteristics of initial subjects and the results of normal and HFFD dietary food intervention.

Characteristic	Normal (mean ± SD)	HFFD (mean ± SD)	p-value
Body weight (g)	246 ± 19.11	245.58 ± 22.06	0.945
Intake (g)	12.23 ± 1.67	6.49 ± 1.52	0.000
Intake fiber (g)	0.35 ± 0.05	0.16 ± 0.04	0.000
Intake (calories from drinks)	0	31.71 ± 7.08	0.000
Intake (calories from diet)	51.60 ± 7.04	34.59 ± 8.12	0.000
Total intake (calories)	51.60 ± 7.04	66.30 ± 7.26	0.000
Final weight (g)	261.93 ± 29.30	279.42 ± 33.22	0.140
Lee index	265.85 ± 10.15	287.65 ± 10.34	0.875
Abdominal circumference (cm)	14.64 ± 0.93	15.84 ± 0.41	0.009

Source: Sulistyowati et al. [21].

**Table 2:** Concentrations of SCFA in rat cecal digesta by group.

Characteristic	N	HFFD	p-value
Acetate (mMol/g)	22.76 ± 6.68	18.18 ± 6.93	0.041
Butyrate (mMol/g)	3.23 ± 1.54	2.72 ± 1.28	0.004
Propionate (mMol/g)	7.31 ± 2.81	5.26 ± 2.09	0.040
Total SCFA (mMol/g)	33.30 ± 8.93	26.16 ± 9.94	0.013

**Table 3:** Pearson’s correlation coefficient between waist circumference and SCFA concentration of cecal digesta and between Lee index and SCFA concentration of cecal digesta.

	Waist circumference	Lee index
SCFA	−0.529*	−0.204

* Significant at α = 0.05.
